# Mixed-Effects Modelling of the Risk Factors Associated with Multiple Pregnancies in Thoroughbred Mares

**DOI:** 10.3390/ani12141841

**Published:** 2022-07-20

**Authors:** Shebl E. Salem, Alannah Sinnott, Jessica M. Roach, Kristien L. P. Verheyen, Amanda M. de Mestre

**Affiliations:** 1 Department of Pathobiology and Population Sciences, The Royal Veterinary College, Hawkshead Lane, Hertfordshire AL9 7TA, UK; s.e.shebl@gmail.com (S.E.S.); kverheyen@rvc.ac.uk (K.L.P.V.); 2 Department of Comparative Biomedical Sciences, The Royal Veterinary College, Hawkshead Lane, Hertfordshire AL9 7TA, UK; asinnott5@rvc.ac.uk (A.S.); jroach@rvc.ac.uk (J.M.R.)

**Keywords:** multiple pregnancies, multiple ovulations, twinning, equine, thoroughbred, broodmare, prostaglandin F2 alpha

## Abstract

**Simple Summary:**

Multiple pregnancies (MPs), or twins/triplets, are commonly diagnosed in pregnant mares. Whilst some studies have identified factors associated with an increased risk of MPs, they have only looked at each factor individually and not accounted for the effects of factors when they occur together. This study used statistical modelling to identify risk factors for MPs, looking at 27 factors as well as the contribution of the mare, stallion (both proxies for genetics), farm, and veterinarian. We found that multiple ovulations and the use of a drug that mimics prostaglandin F2α to induce oestrus, both increased the risk of a mare having an MP. Mares that had a foal that same year, had a uterine cyst, or who did not get pregnant on the first cycle they were bred on were at a decreased risk of having an MP. Factors that impact the early embryonic environment are more important influences of multiple pregnancies when compared to the genetics of the mare. The increased incidence of MP but not MOs over the previous decades may well reflect improved management of the endometrium as opposed to selection of mares with increased risk for MPs. A limitation of the study is the reliance on clinical records being completed accurately.

**Abstract:**

Multiple pregnancies (MPs) are commonly diagnosed during breeding management of mares. Whilst some studies have reported on factors associated with the risk of MPs, few have utilised multivariable data analysis to control for confounding variables. A prospective cohort study of Thoroughbred broodmares was conducted with information collected on 27 factors. Mixed-effects logistic regression was used to determine risk factors for MPs. Mare, stallion, stud, and veterinarian were evaluated as random effects. The prevalence of MPs in 1754 mares and 2245 pregnancies was 16.06% (95% confidence interval [CI] = 14.54, 17.58). Multiple ovulations (OR = 15.57, 95% CI = 11.88, 20.53) and treatment with cloprostenol (OR = 1.35, 95% CI = 1.015, 1.80) were associated with increased odds of MPs following multivariable analysis. Mares that foaled at the start of the breeding season (OR = 0.66, 95% CI = 0.47, 0.94), conceived at the second or more oestrus cycles (OR = 0.60, 95% CI= 0.43, 0.84), or identified with a uterine cyst (OR = 0.63, 95% CI = 0.40, 0.97) were at reduced odds of conceiving MPs. Mare, stallion, stud, and veterinarian were not associated with MPs. These findings provide possible explanations as to why the prevalence of MPs but not MOs have increased over the last decade.

## 1. Introduction

Multiple pregnancies (MPs) in mares have long been recognised as a major cause of abortion, stillbirth, or birth of foals that have reduced chances of survival [1,2]. Multiple pregnancies in horses are mainly heterozygotic, a direct result of multiple ovulations, and fertilisation of multiple follicles [3], with monozygotic twins [4] or triplets [5] extremely rare after natural breeding in mares. They have been more often associated with pregnancies established through embryo transfer [6], although they still remain uncommon, with monozygotic multiple pregnancies reported in just 1.6% of pregnancies following transfer of in vitro-produced embryos [7]. Natural elimination of MPs to singletons has been reported, especially when the conceptuses are dissimilar in size and fixed in the same location in the uterus likely mediated via a compromised trilaminar yolk-sac [8]. Nevertheless, in routine clinical practice, manual reduction of MPs is applied as a standard procedure to prevent the MPs developing into the foetal stage, imposing risk on both the foetuses and the mare [2]. The procedure is most successful before the period of embryonic fixation (16–17 gestation days), as the conceptuses can be easily separated and reduced by minimal pressure of the hand of the operator or of the ultrasound probe [9]. 

Knowledge of the factors associated with increased risk of MPs can assist with identifying mares at risk. Furthermore, the incidence of MPs could be reduced if modifiable risk factors are identified and used to inform preventive measures. Previously, mares that had foaled at the start of the breeding season have been reported to be at lower risk of developing MPs [10,11,12]. Age of mares was not found to be a determinant of the incidence of MPs in a number of studies [10,11,13]. Mares that had oestrus/ovulation induction through administration of prostaglandin F2α, human chorionic gonadotropin (hCG), or both, were also found to be at greater risk of developing either multiple ovulations and/or MPs [13]. Treatment with hCG in another study from New Zealand was associated with a three-fold increase in the likelihood of developing MPs [12]. Similar findings were also reported by Allen, Brown, Wright, and Wilsher [11], where mares that had induced ovulations carried significantly greater number of MPs compared with mares that were allowed to ovulate spontaneously. 

The prevalence of MPs reported in Thoroughbreds between 1993 and 2018 has shown a gradual increase over time. Whether this is due to genetic reasons, notable changes in the use of reproductive hormones in stud medicine [14,15], or other as yet unidentified reasons is not known. Apart from the study conducted by Perkins and Grimmett [12], which investigated risk factors for MPs in Thoroughbred farms in the Waikato region of New Zealand, none of the previous studies (reviewed above) have used multivariable analysis techniques to control for potential confounding variables when investigating the risk of MPs. Furthermore, none of these studies have investigated the influence of breeding stallion, stud, or veterinarian (proxies for blood lines and breeding management) on the incidence of MPs in the mare. Therefore, the objectives of the current study were to use multivariable analysis techniques to investigate risk factors for MPs in Thoroughbred mares and to estimate the amount of variation in the risk of MPs that could be explained by mare, breeding stallions, stud farm, or veterinarian. 

## 2. Materials and Methods

### 2.1. Study Design

A prospective cohort study was primarily conducted to investigate risk factor for early pregnancy loss (EPL) in Thoroughbred mares [14,16]. The study collected reproductive information on 2245 pregnancies in 1754 mares over the 2013 and 2014 breeding seasons. The mares were recruited from 32 studs in and around Suffolk, United Kingdom, and were under the veterinary care of two equine practices. A multiple pregnancy was defined as presence of two or more embryonic vesicles/foetuses during reproductive ultrasound examination at any point during pregnancy. Sample size calculations for a cohort study showed that around 628 pregnancies would be required to identify 50% reduction in the odds of MPs in mares foaled at the start of the breeding season compared with maiden, barren, or rested mares, assuming 19% prevalence of multiple pregnancies in the unexposed group [10] at a study power of 80% and 95% confidence level. Sample size calculations were performed using Epi Info 7 software (Centre for Disease Control, Atlanta, GA, USA).

### 2.2. Data Collection and Processing

Breeding and reproductive data were collected at the time of the initial positive pregnancy ultrasound scan 11–15 days post covering. Information was collected using a data collection form as previously described [14]. The form included questions about the mare (age, breeding status at the start of the season, any previous EPL, number of previous breeding seasons (number of seasons a mare was bred to a stallion with or without conception) and live foals); pre-oestrus, pre-ovulation, and post ovulation medications; number of oestrus cycles before a positive pregnancy scan; presence of uterine cyst; presence of uterine fluid; number of ovulations and number of embryonic vesicles; date of covering and the covering stallion. Findings on subsequent ultrasound scans until gestation day 65 were also recorded. Additional information was collected from the veterinary practices databases, Weatherbys Return of Mares Annual Reports (Wellingborough, UK) and Racing Post online (https://www.racingpost.com/bloodstock/ (accessed on 1 February 2017)). All the data were anonymised and entered into a custom-built Microsoft Access database. 

### 2.3. Statistical Analysis

Data tables from the Microsoft Access database were imported into R software and joined using the dplyr package [17]. The prevalence of MPs, together with the Wald 95% confidence interval (CI), was calculated for different levels of each exposure variable. Two-level random intercept logistic regression was performed to identify factors associated with MPs, and odds ratios (OR) and their 95% CI reported. Mare was included as a random effect in all analyses (level 2), to account for repeat pregnancies (level 1) in the same mare within the dataset. The models were fitted using the glmer::lme4 function in R where the adaptive Gauss–Hermite approximation to the log-likelihood is used [18]. The functional form (shape) of the relationship between continuous predictor variables and the log odds of MPs was explored using generalised additive models [19]. The outputs from the models were plotted and if departure from linear trend was evident, the variable was categorised using data driven cut-off points before inclusion into logistic regression analysis. A total of 27 exposure variables were evaluated as fixed effects in univariable analyses (Supplementary Table S1).

Variables with a univariable likelihood ratio test (LRT) *p*-value of < 0.2, were considered for building a multivariable two-level mixed effects logistic regression model. Twenty-five variables had less than 3.3% missing observations (Supplementary Table S1). Two variables; EPL at previous season and number of days from foaling to covering were excluded from model building as they contained greater than 20% missing observations (Supplementary Table S1). The model was built using a forward stepwise approach where inclusion of a variable in the final model was based on a LRT *p*-value of < 0.05 when nested models were compared. Biologically plausible interactions between variables in the final model were assessed using the LRT comparing models with and without the interaction terms. Stud farm, stallion, and veterinarian were assessed as random effects both in the null (with no other variables included) and in the final full model. Models with and without the random effects were compared using the LRT. Before testing for the random effects, we excluded studs, stallions and veterinarian that contributed fewer than five observations. Excluded variables that met the *p* < 0.2 criteria in univariable analysis were forced back into the model to check they did not reach statistical significance when adjusted for the other variables in the model or confounded other associations. The random effect of mare contributed a close to zero variance in the final full multivariable model and therefore it was excluded. The model was then evaluated for goodness-of-fit using the Hosmer–Lemeshow goodness-of-fit test using 10 groups divided by deciles of fitted values. Influential observations were identified, and the model was re-run following exclusion of each to test for their leverage on parameter estimates. Collinearity between variables retained in the final model was investigated by calculation of generalised variance-inflation factor for each of them using the car::vif function in R. Statistical analyses were performed using R software version 3.5.3 [20].

## 3. Results

### 3.1. Descriptive Results

The study collected information on 2245 pregnancies occurring in 1754 mares over two breeding seasons. There were 411 mares that were bred in both seasons and 80 mares that had repeat pregnancies within the same season resulting in a total of 491 repeat pregnancies in the dataset. The rest of the mares were bred in only one of the breeding seasons (777 in 2013, 566 in 2014). The mares were recruited from 32 stud farms, covered by 86 stallions and veterinary care was provided by 13 veterinarians. The mares had a median age of 8 years (IQR = 5, 11) and exhibited a median of 1.3 oestrus cycles before a positive pregnancy scan was confirmed (IQR = 1, 2). Four mares did not have information on the number of conceived pregnancies and were excluded from further analysis. 

The prevalence of MPs in this cohort was 16.1% (360/2241, 95% CI = 14.5, 17.6). Of these, 344 mares carried twins and 16 mares carried triplets. Location of twin pregnancies at the time of manual reduction was recorded for 253 mares. Of these, 95 mares had a unilateral twin pregnancy, and 158 mares had a bilateral twin pregnancy. Location of triplet pregnancies was recorded for 12 mares, of which 4 mares had unilateral triplet pregnancies and 8 mares had two vesicles at the same location in the uterus and a third at a different location. Multiple pregnancies were manually reduced at a median of 16 days (IQR = 15, 16) with 59.3% (211/356) of the mares being administered flunixin meglumine (Cronyxin^®^, Bimeda, Anglesey, UK, Flunixin^®^, Norbrook), 2.2% (8/356) receiving hyoscine butylbromide (Buscopan^®^, Boehringer, Bracknell, UK) and 24.4% (87/356) being sedated during the procedure. 

### 3.2. Univariable Analysis

**Figure 1:**
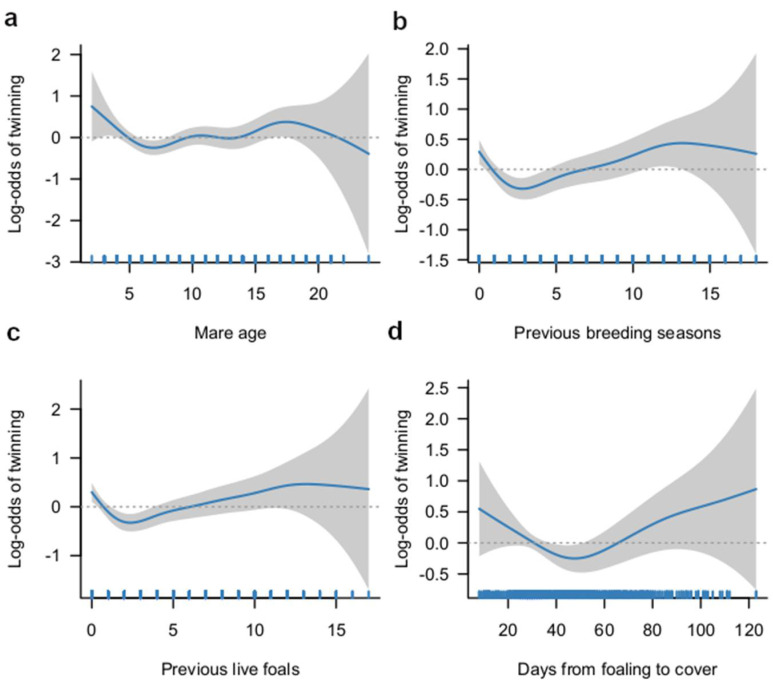
Graphical illustrations of the estimated relationships (‘the smooth’) between the log-odds of multiple pregnancies and: (**a**) age of the mares; (**b**) number of previous breeding seasons; (**c**) number of previous live foals; and (**d**) days from foaling to cover. The plots show fitted curves (blue lines) with 95% confidence intervals (the gray shading). Rug plots along the *x*-axes represent the number of data points. The dotted horizontal lines are at log-odds = 0.

A summary of the 27 exposure variables investigated, categories for each variable, prevalence of MPs for each category, and results of univariable logistic regression analyses are presented in Supplementary Table S1. Non-linear relationships between the likelihood of MPs and age of the mares, number of previous breeding seasons, number of previous live foals, and number of days from foaling to covering were identified (Figure 1) and these variables were therefore evaluated as categorical variables in the logistic regression analysis. Variables that were significantly (LRT < 0.05) associated with the odds of MP in univariable analysis were: breeding status at the start of the season, EPL at the previous breeding season, number of previous live foals, number of previous breeding seasons, number of oestrus cycles before a positive pregnancy scan, month of covering, pre-oestrus prostaglandin F2α analogue administration (cloprostenol), any pre-oestrus hormone treatment, multiple ovulations, and uterine cysts identified on ultrasound examination. These variables together with two other variables with an LRT *p*-value < 0.2 (pre-oestrus gonadotrophins treatment and ovulatory medications) were taken forward to build a multivariable mixed-effects logistic regression model. 

### 3.3. Multivariable Analysis

The results of the final multivariable model are presented in Table 1. Mares that had foaled at the start of the breeding season, were covered at two or more oestrus cycles before a positive pregnancy scan or identified with a uterine cyst were at reduced odds of carrying an MP. Multiple ovulations and prostaglandin F2α analogue (cloprostenol) treatment were associated with increased odds of an MP. There were no significant interaction terms identified in the final model. The Hosmer–Lemeshow test statistic suggested that the model was a good fit (*p*-value = 0.79). Four influential observations were identified; however, they had a minimal effect on the coefficients in the model and therefore they were retained in the final model. None of the random effects tested either in the null or in the full final multivariable model explained a significant amount of variation in the occurrence of MPs in this cohort of mares as indicated by LRT (Table 2). Caterpillar plots of residuals associated with stud farm, mare, stallion, or veterinarian random-effects are presented in Supplementary Figure S1.

## 4. Discussion

This study identified five risk factors associated with a modified risk of MPs. Pre-oestrous treatment with prostaglandin F2α analogue led to a small increased risk of MPs (1.4 times). Three factors, the mare having foaled earlier in the breeding season, the mare conceiving on her second or subsequent oestrous cycle that she was bred on, and the presence of a uterine cyst in the mare, were all associated with a small but significantly decreased risk of MPs (1.5 to 1.7 times). As expected, the factor which had the greatest influence on MP was multiple ovulation (15.6 times). Contrary to some previous studies, no significant variance in MP risk was identified for mare age and hCG or through inclusion of the mare, stallion, stud, nor veterinarian in either the empty or final models.

The prevalence of MPs (16.06%) reported in the current study exceeds previously reported rates of MPs in Thoroughbred stud farms in New Zealand (12.9–13.3%) [12,21], north-east Victoria (12.1%) [22], south eastern Australia (7.8%) [23], Sweden (10.5%) [24], and the UK (13.1%) [11]. This discrepancy in the prevalence of MPs between the current and other studies could be due to variation in study design, e.g., a prospective vs. a retrospective design, improvement in diagnostic imaging technologies that has led to more accurate diagnosis of MPs, or better veterinary management of oestrus cycle over time with subsequent general improvement in the fertility of Thoroughbred mares [10,11]. In support of improved fertility, the prevalence of multiple ovulations in our cohort of mares (22%) is comparable to previously reported rates (22.4%) [25]. It may also reflect the characteristics of the cohorts under study such as variations in the number of foaling mares and the increased use of therapeutics such as PGF2 α analogues, both found here to modify risk of MPs.

The only therapeutic found to modify risk of conceiving an MP was PGF2 α analogue cloprostenol. Whether this is directly leading to MPs is not known. Luprostol, another Prostaglandin analogue, has been found to cause a surge in LH and FSH within 2 to 10 min in the intercavernous sinus and jugular vein of mares, with these gonadotrophins not returning to baseline to 240 min after administration. This is a possible mechanism by which PGF2 α analogues could therefore impact follicle development [26]. Further, Veronesi et al. 2003 has showed an increased risk for multiple ovulations associated with PGF2 α. Here we found no modified risk of MP associated with hCG or deslorelin when used as ovulatory induction agents suggesting that it was primarily the PGF2 α not the hCG that was responsible for the observed increased incidence of MP by Veronesi et al., 2003. Further studies are required to determine the exact mechanism by which PGF2 α analogues may be acting to modify the risk of ovulation of multiple follicles or alternatively to determine whether it may be acting on improving the quality of the early embryonic environment, notably the endometrium.

Whilst we found no association between deslorelin and MP, it should be noted that deslorelin, when administered twice daily in early oestrus, has been found to increase the number of double ovulations and median number of embryos recovered when compared with saline-treated mares [27]. This treatment protocol was not assessed in this study. We also know that hCG and deslorelin reduce the risk of early pregnancy loss which could plausibly be attributed to an improved early embryonic environment as opposed to an effect on follicle number or quality. Collectively, these observations demonstrate the complexity of the effects of deslorelin on multiple ovulations and MP as well as pregnancy maintenance and care should be taken to consider the dose, protocol, and confounding factors in any future interventional studies that aim to assess these outcomes.

In the current study, mares that foaled at the start of the breeding season had 47% lower odds of carrying MPs. This finding has been consistently reported in published research [10,11,12,21]. Biologically, foaling mares would have had naturally higher metabolic demands at the time of breeding due to their status; having been bred during foal heat, energy requirements are higher due to lactation as well as having recently foaled. Restricted intake of food in non-lactating mares has been associated with reduced total number of follicles as well as higher FSH concentrations in follicular fluid [28]. It is plausible that similar mechanisms act in lactating mares due to redirection of nutrients to lactation. Alternatively, it may be that within foaling mares, less nutrients and energy are available for supporting the early embryonic environment and conception of MPs. It might also have been that non-foaling mares were more likely to have received a form of pre-oestrus hormone treatment in comparison to foaling mares to improve their chances of conceiving a pregnancy, which might have impacted on the risk of MPs. However, in our analysis, foaling status remained a significant risk factor after controlling for other variable such as pre-oestrous prostaglandin analogue and multiple ovulations.

Multiple ovulation was overwhelmingly the greatest influence on risk for MPs in this study (15.6 times), consistent with the rarity of monozygotic multiple pregnancies [7]. The remaining two variables (uterine cysts, covering on multiple cycles) were both associated with a modest reduced risk of MPs and likely are a reflection on the ability of the endometrium to support an MP. Endometrial cysts have been associated with an increased risk of pregnancy loss between day 15 and 65 of gestation [16]. Studies suggest that cysts may reduce blood perfusion within uterine vasculature [29] which could compromise nutrients and energy supply to the developing embryo collectively required for early development. Cysts could also be a proxy for a compromised wider quality of the endometrium which would similarly reduce embryonic survival prior to the first pregnancy diagnosis. Covering of a mare on multiple cycles reflects either that she failed to conceive or previously failed to carry an embryo to day 15. Similarly to cysts, it is likely this variable reflects a suboptimal endometrial environment. Also of note was the lack of association between mare age and MPs, in contrast to the association found between increased mare age and multiple ovulation [30]. This may reflect failure of fertilisation of ova or again reflect the increased risk of EPL in aged mares [16]. Indeed, a previous study found older mares were at decreased risk of MPs [25].

Mare, stallion, and farm were not found to be associated with MPs when included in the final model. Whilst stallion and farm are not unexpected findings, based on the observation that mares that previously had a multiple ovulation were at greater risk of having a multiple ovulation in successive cycles [25], we were surprised not to identify mare as a contributor to variance. One explanation could be that the mare variance identified in previous studies was accounted for by the mare-related variables included in our models such as uterine cysts, status, and number of cycles covered. Furthermore, it is a reminder that MPs are not only a reflection on multiple ovulations but also the fertility of the mare and, importantly, the quality of the embryonic environment prior to pregnancy diagnosis.

## 5. Conclusions

There has been a small increase in the prevalence of MPs in TB mares over the last two decades. Previously, it has been speculated that the increase in MPs is driven by selection of mares with a genetic predisposition to MOs, but here we did not find any evidence to support this with mare not contributing to variance in this study. This suggests that whilst mare might contribute to variance associated with MO, this does not automatically apply to MPs. An alternative explanation for an increase is MPs is the increased use of PGF2 α analogues in practice, found here to increase the risk of MPs. Further, the decreased risk associated with multiple covers and uterine cysts supports the important role for the early embryonic environment in supporting progressing of embryos to at least 15 days of gestation. Given the significant efforts over the last decade to prepare the endometrium for conception, it is also plausible that the increase in MPs can be attributed to improved conception rates irrespectively of one or multiple embryos being present.

## Figures and Tables

**Table 1:** Multivariable logistic regression model of risk factors for multiple pregnancies in a cohort of 1754 Thoroughbred mares over the 2013 and 2014 breeding seasons (*n* = 2241 pregnancies). The mare random effect was excluded from the model, as the mare-level variance in the occurrence of multiple pregnancies was close to zero.

Variable	Level	Coefficient	Standard Error	Odds Ratio (OR)	95% Confidence Interval	Wald Test *p*-Value	Likelihood Ratio Test *p*-Value
Foaling status	Maiden	Reference					0.03
	Barren	0.012	0.22	1.01	0.65, 1.56	0.96	
	Foaled	−0.41	0.18	0.66	0.47, 0.94	0.02	
	Rested	0.08	0.33	1.08	0.56, 2.03	0.81	
Number of cycles covered	1	Reference					0.003
	≥2	−0.50	0.17	0.60	0.43, 0.84	0.003	
Uterine cysts	No	Reference					0.04
	Yes	−0.46	0.22	0.63	0.40, 0.97	0.04	
Multiple ovulation	No	Reference					<0.001
	Yes	2.75	0.14	15.57	11.88, 20.53	<0.001	
Pre-oestrus prostaglandin F2α treatment	No	Reference					0.04
	Yes	0.30	0.15	1.35	1.02, 1.80	0.04	

**Table 2:** Variances of the random effect variables when included in null and in the full final multivariable models and the likelihood ratio test *p*-values comparing models with and without the random effect variables. Models with more than one random effect did not converge.

Random Effect	Variance in the Null Model	Likelihood Ratio Test *p*-Value	Variance in the Full Final Model	Likelihood Ratio Test *p*-Value
Stud	0.002	0.93	0.02	0.92
Mare	0.58	0.15	<0.001	0.90
Breeding Stallion	0.01	0.77	0.01	0.93
Veterinary Surgeon	0.01	0.58	0.06	0.99

## Data Availability

The data presented in this study are available on request from the corresponding author. The data are not publically available due to confidentiality.

## Supplementary Materials

The following supporting information can be downloaded at: https://www.mdpi.com/article/10.3390/ani12141841/s1, Table S1: Descriptive and univariable analysis results of potential risk factors for multiple pregnancies (twinning) in a cohort of 1754 thoroughbred mares (2245 pregnancies) in the UK in the 2013 and 2014 breeding seasons. ‘Mare’ was included in the models as a random effect to account for repeat pregnancies in the same mare. Figure S1: The plots show the estimated residuals for the random-effects of (**a**) studs; (**b**) mares; (**c**) stallions; and (**d**) veterinarians from the null models. For all variables, the 95% confidence interval does overlap the horizontal line at zero, indicating that the risk of multiple pregnancies did not differ significantly between studs, mares, stallions, or veterinarians. Only stud farms that contributed ≥ 5 mares and stallions that mated ≥ 5 mares were included in this analysis.
