# Supplementary material for: Mixed-Effects Modelling of the Risk Factors Associated with Multiple Pregnancies in Thoroughbred Mares
Source: Animals (Basel). 2022 Jul 20;12(14):1841. doi: 10.3390/ani12141841 (PMC9312083; doi:10.3390/ani12141841)

Table S1: Descriptive and univariable analysis results of potential risk factors for multiple pregnancies (twinning) in a cohort of 1,754 thoroughbred mares (2,245 pregnancies) in the UK in the 2013 and 2014 breeding seasons. 'Mare' was included in the models as a random effect to account for repeat pregnancies in the same mare.

**Variable**	**Level**	**n**	**MP**	**MP %**	**95% confidence interval (MP prevalence)**	**β**	**Standard error (β)**	**Odds ratio (OR)**	**95% confidence interval (OR)**	**Likelihood ratio test p-value**	**% missing values**
Mare age (n=2239)	2-5	574	103	17.94	14.81, 21.08	Ref.				0.06	0.27
	6-10	976	135	13.83	11.67, 16.00	-0.34	0.16	0.71	0.53, 0.97		
	11+	689	119	17.27	14.45, 20.09	-0.06	0.16	0.94	0.69, 1.30		
Mare status (n=2245)	Maiden	416	90	21.63	17.68, 25.59	Ref.				<0.001	0
	Barren	308	71	23.05	18.35, 27.76	0.08	0.19	1.08	0.74, 1.58		
	Foaled	1407	177	12.58	10.85, 14.31	-0.69	0.16	0.50	0.37, 0.68		
	Rested	114	22	19.30	12.05, 26.54	-0.14	0.28	0.87	0.50, 1.51		
EPL previous season (n=1541)	No	1422	224	15.75	13.86, 17.65	Ref.				0.03	31.36
	Yes	119	28	23.53	15.94, 31.15	0.59	0.27	1.81	1.06, 3.10		
Previous live foals (n=2206)	0	469	101	21.54	17.81, 25.26	Ref.				<0.001	1.74
	1-6	1187	151	11.82	10.83, 14.62	-0.68	0.16	0.51	0.37, 0.69		
	7+	550	101	18.36	15.13, 21.60	-0.21	0.17	0.81	0.58, 1.14		
Previous breeding seasons (n= 2207)	0	415	89	21.45	17.50, 25.39	Ref.				<0.001	1.7
	1-7	1375	185	13.45	11.65, 15.26	-0.61	0.16	0.54	0.40, 0.74		
	8+	417	79	18.94	15.18, 22.71	-0.16	0.19	0.85	0.58, 1.24		
Number of cycles covered (n=2234)	1	1655	290	17.52	15.69, 19.35	Ref.				0.002	0.5
	≥2	455	70	12.09	9.43, 14.75	-0.47	0.16	0.63	0.46, 0.85		
Days from foaling to cover (n=1346)	8-35	817	109	13.34	11.01, 15.67	Ref.				0.03	40.04
	36-65	414	37	8.94	6.19, 11.69	-0.50	0.23	0.61	0.39, 0.94		
	66-123	113	18	15.93	9.18, 22.68	0.28	0.32	1.32	0.70, 2.48		

Variable	Level	n	MP	MP %	95% confidence interval (MP prevalence)	β	Stan dard error (β)	Odds ratio (OR)	95% confidence interval (OR)	Likelihood ratio test p-value	% missing values
Month of cover (n=2243)	Feb	304	67	22.04	17.38, 26.70	Ref.				0.02	0.09
	March	677	113	16.69	13.88, 19.50	-0.36	0.19	0.70	0.48, 1.01		
	April	672	96	14.29	11.64, 16.93	-0.57	0.20	00.56	0.38, 0.83		
	May	514	76	14.79	11.72, 17.83	-0.53	0.21	0.59	0.39, 0.88		
	June-August	76	8	10.53	3.63, 17.43	-0.97	0.43	0.38	0.16, 0.88		
Uterine fluid (n=2202)	No	1443	229	15.87	13.98, 17.75	Ref.				0.64	1.92
	Yes	759	127	16.73	14.08, 19.39	0.06	0.13	1.06	0.82, 1.37		
Uterine fluid (cm) (n=2202)	0	1443	229	15.87	13.98, 17.75	Ref.				0.79	1.92
	1	532	85	15.98	12.86, 19.09	0.00	0.15	1.00	0.75, 1.34		
	2	147	28	19.05	12.70, 25.40	0.24	0.24	1.27	0.79, 2.03		
	≥3	80	14	17.50	9.17, 25.83	0.12	0.33	1.12	0.59, 2.13		
Uterine cysts (n=2240)	No	1916	325	16.96	15.28, 18.64	Ref.				0.004	0.22
	Yes	324	35	10.8	7.42, 14.18	-0.56	0.2	0.57	0.38, 0.85		
Multiple ovulation (n=2215)	No	1728	108	6.25	5.11, 7.39	Ref.				<0.001	1.34
	Yes	487	248	50.92	46.48, 55.36	2.74	0.13	15.56	11.95, 20.24		
Multiple ovulations (n=2215)	None	1728	108	6.25	5.11, 7.39	Ref.				<0.001	1.34
	Unilateral	206	101	49.03	42.20, 55.86	2.67	0.17	14.42	10.31, 20.17		
	Bilateral	281	147	52.31	46.47, 58.15	2.8	0.15	16.46	12.13, 22.30		
Pre-oestrus Cloprostenol (n=2181)	No	1534	220	14.34	12.59, 16.10	Ref.				0.001	2.85
	Yes	647	131	20.25	17.15, 23.34	0.45	0.14	1.56	1.19, 2.04		
Pre-oestrus altrenogest (n=2181)	No	2044	328	16.05	14.46, 17.64	Ref.				0.83	2.85
	Yes	137	23	16.79	10.53, 23.05	0.06	0.26	1.06	0.64, 1.76		
Pre-oestrus domperidone (n=2180)	No	2121	343	16.10	14.54, 17.66	Ref.				0.97	2.9
	Yes	49	8	16.33	5.98, 26.6	0.016	0.42	1.02	0.44, 2.33		

Variable	Level	n	MP	MP %	95% confidence interval (MP prevalence)	β	Standard error (β)	Odds ratio (OR)	95% confidence interval (OR)	Likelihood ratio test p-value	% missing values
Pre-oestrus sulpiride (n=2180)	No	2122	344	16.21	14.64, 17.78	Ref.				0.38	2.9
	Yes	58	7	12.07	3.69, 20.45	-0.37	0.44	0.69	0.29, 1.63		
Pre-oestrus domperidone or sulpiride (n=2174)	No	2068	334	16.15	14.56, 17.74					0.57	3.16
	Yes	106	15	14.15	7.52, 20.79	-0.17	0.31	0.84	0.46, 1.54		
Pre-oestrus GNRH (n = 2245)	No	2209	357	16.16	14.63, 17.70	Ref.				0.19	0
	Yes	36	3	8.33	-0.70, 17.36	-0.77	0.64	0.47	0.13, 1.62		
Any pre-oestrus progesterone (n=2245)	No	2029	319	15.72	14.14, 17.31					0.22	0
	Yes	216	41	18.98	13.75, 24.21	0.25	0.20	1.28	0.87, 1.90		
Any pre-oestrus hormones (n=2232)	No	1370	196	14.31	12.45, 16.16	Ref.				0.003	0.58
	Yes	862	164	19.03	16.41, 21.65	0.37	0.13	1.44	1.13, 1.85		
Ovulatory induction agent (n=2190)	No	174	22	12.64	7.71, 17.58	Ref.				0.18	2.49
	Yes	2016	329	16.32	14.71, 17.93	0.34	0.26	1.40	0.84, 2.32		
Ovulatory induction agent (n=2190)	None	174	22	12.64	7.71, 17.58	Ref.				0.08	2.49
	HCG	591	83	14.04	11.24, 16.85	0.14	0.28	1.15	0.66, 2.00		
	Deslorelin	1407	240	17.06	15.09, 19.02	0.40	0.26	1.49	0.89, 2.50		
	Other*	17	5	29.41	7.75, 51.07	1.22	0.67	3.39	0.92, 12.54		
Intrauterine antibiotics at cover (n=2186)	No	1092	170	15.57	13.42, 17.72	Ref.				0.61	2.63
	Yes	1094	179	16.36	14.17, 18.55	0.07	0.13	1.07	0.83, 1.37		
Dexamethasone at cover (n=2186)	No	2055	328	15.96	14.38, 17.54	Ref.				0.99	2.63
	Yes	131	21	16.03	9.75, 22.31	-0.00	0.27	0.99	0.59, 1.69		
Oxytocin at cover (n=2185)	No	1081	174	16.10	13.91, 18.29	Ref.				0.80	2.67
	Yes	1104	174	15.76	13.61, 17.91	-0.03	0.13	0.97	0.75, 1.24		
Intrauterine lavage at cover (n=2186)	No	1668	262	15.71	13.96, 17.45	Ref.				0.52	2.63
	Yes	518	87	16.80	13.58, 20.01	0.1	0.15	1.10	0.82, 1.47		

*Other ovulatory medications: Busorelin (n = 3), HCG + Busorelin (n = 10), HCG + Deslorelin (n = 1), ECG (n = 2), HCG + Deslorelin + Busorelin (n = 1)

Figure S1: The plots show the estimated residuals for: (**a**) studs; (**b**) mares; (**c**) stallions; and (**d**) veterinarians random-effects from the null models. For all variables, the 95% confidence interval does overlap the horizontal line at zero, indicating that the risk of multiple pregnancies did not differ significantly between studs, mares, stallions, or veterinarians. Only stud farms that contributed ≥ 5 mares and stallions that mated ≥5 mares were included in this analysis.

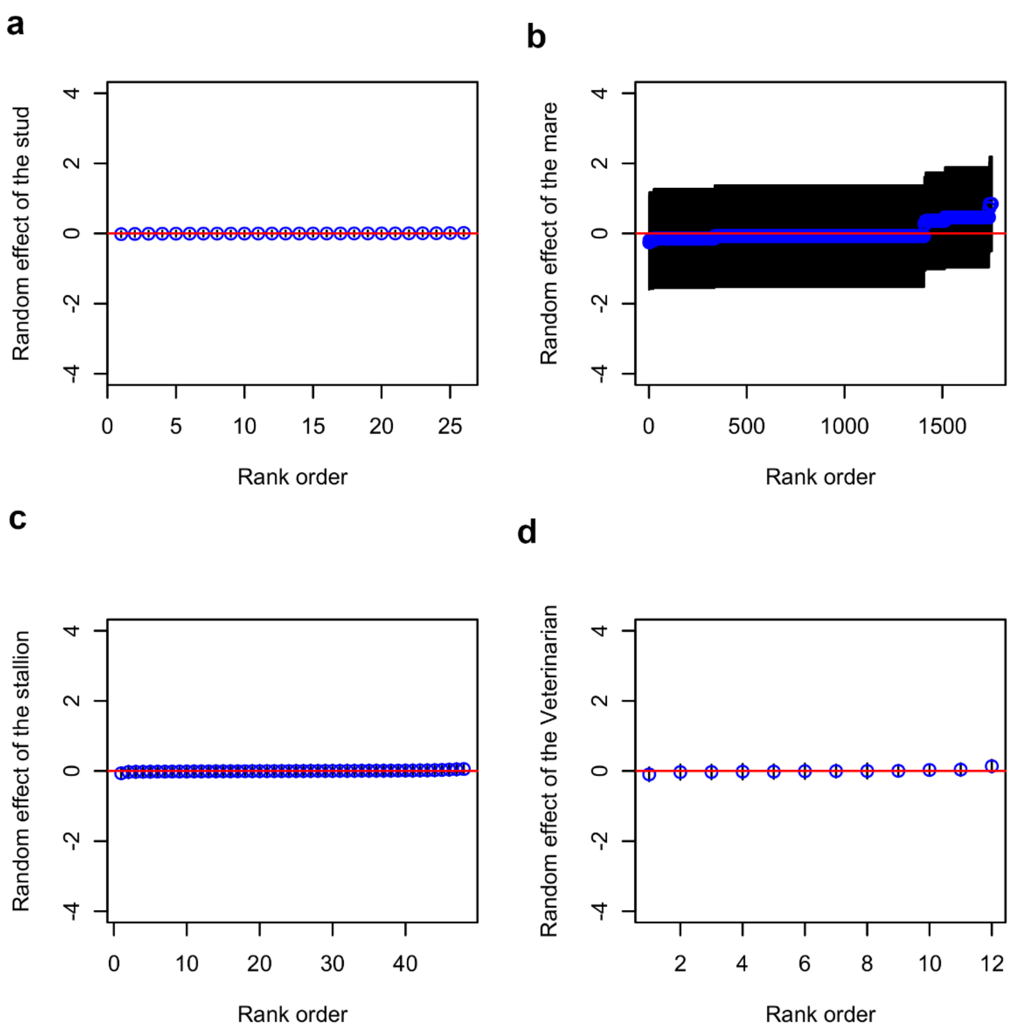

Supplement: Supplementary file 1 [file animals-12-01841-s001.zip › animals-1798175-supplementary.pdf]
